# Integrated Analysis of Patient Networks and Plasmid Genomes to Investigate a Regional, Multispecies Outbreak of Carbapenemase-Producing Enterobacterales Carrying Both *bla*_IMP_ and *mcr-9* Genes

**DOI:** 10.1093/infdis/jiae019

**Published:** 2024-01-20

**Authors:** Yu Wan, Ashleigh C Myall, Adhiratha Boonyasiri, Frances Bolt, Alice Ledda, Siddharth Mookerjee, Andrea Y Weiße, Maria Getino, Jane F Turton, Hala Abbas, Ruta Prakapaite, Akshay Sabnis, Alireza Abdolrasouli, Kenny Malpartida-Cardenas, Luca Miglietta, Hugo Donaldson, Mark Gilchrist, Katie L Hopkins, Matthew J Ellington, Jonathan A Otter, Gerald Larrouy-Maumus, Andrew M Edwards, Jesus Rodriguez-Manzano, Xavier Didelot, Mauricio Barahona, Alison H Holmes, Elita Jauneikaite, Frances Davies

**Affiliations:** NIHR Health Protection Research Unit in Healthcare Associated Infections and Antimicrobial Resistance, Department of Infectious Disease, Imperial College London, London, United Kingdom; NIHR Health Protection Research Unit in Healthcare Associated Infections and Antimicrobial Resistance, Department of Infectious Disease, Imperial College London, London, United Kingdom; Department of Mathematics, Imperial College London, London, United Kingdom; NIHR Health Protection Research Unit in Healthcare Associated Infections and Antimicrobial Resistance, Department of Infectious Disease, Imperial College London, London, United Kingdom; Faculty of Medicine, Siriraj Hospital, Mahidol University, Bangkok, Thailand; Department of Infectious Diseases, Imperial College Healthcare NHS Trust, London, United Kingdom; Centre for Antimicrobial Optimisation, Hammersmith Hospital, Imperial College London, London, United Kingdom; Department of Infectious Disease Epidemiology, School of Public Health, Imperial College London, London, United Kingdom; Department of Infectious Disease Epidemiology, School of Public Health, Imperial College London, London, United Kingdom; HCAI, Fungal, AMR, AMU and Sepsis Division, UK Health Security Agency, London, United Kingdom; Department of Infectious Diseases, Imperial College Healthcare NHS Trust, London, United Kingdom; School of Biological Sciences, University of Edinburgh, Scotland, United Kingdom; School of Informatics, University of Edinburgh, Scotland, United Kingdom; NIHR Health Protection Research Unit in Healthcare Associated Infections and Antimicrobial Resistance, Department of Infectious Disease, Imperial College London, London, United Kingdom; HCAI, Fungal, AMR, AMU and Sepsis Division, UK Health Security Agency, London, United Kingdom; NIHR Health Protection Research Unit in Healthcare Associated Infections and Antimicrobial Resistance, Department of Infectious Disease, Imperial College London, London, United Kingdom; Department of Microbiology, North West London Pathology, London, United Kingdom; MRC Centre for Molecular Bacteriology and Infection, Department of Infectious Disease, Faculty of Medicine, Imperial College London, London, United Kingdom; MRC Centre for Molecular Bacteriology and Infection, Department of Infectious Disease, Faculty of Medicine, Imperial College London, London, United Kingdom; Department of Infectious Diseases, Imperial College Healthcare NHS Trust, London, United Kingdom; NIHR Health Protection Research Unit in Healthcare Associated Infections and Antimicrobial Resistance, Department of Infectious Disease, Imperial College London, London, United Kingdom; Centre for Bio-Inspired Technology, Department of Electrical and Electronic Engineering, Faculty of Engineering, Imperial College London, London, United Kingdom; NIHR Health Protection Research Unit in Healthcare Associated Infections and Antimicrobial Resistance, Department of Infectious Disease, Imperial College London, London, United Kingdom; Centre for Bio-Inspired Technology, Department of Electrical and Electronic Engineering, Faculty of Engineering, Imperial College London, London, United Kingdom; Department of Microbiology, North West London Pathology, London, United Kingdom; NIHR Health Protection Research Unit in Healthcare Associated Infections and Antimicrobial Resistance, Department of Infectious Disease, Imperial College London, London, United Kingdom; Department of Infectious Diseases, Imperial College Healthcare NHS Trust, London, United Kingdom; NIHR Health Protection Research Unit in Healthcare Associated Infections and Antimicrobial Resistance, Department of Infectious Disease, Imperial College London, London, United Kingdom; HCAI, Fungal, AMR, AMU and Sepsis Division, UK Health Security Agency, London, United Kingdom; NIHR Health Protection Research Unit in Healthcare Associated Infections and Antimicrobial Resistance, Department of Infectious Disease, Imperial College London, London, United Kingdom; Reference Services Division, UK Health Security Agency, London, United Kingdom; NIHR Health Protection Research Unit in Healthcare Associated Infections and Antimicrobial Resistance, Department of Infectious Disease, Imperial College London, London, United Kingdom; NIHR Health Protection Research Unit in Healthcare Associated Infections and Antimicrobial Resistance, Department of Infectious Disease, Imperial College London, London, United Kingdom; MRC Centre for Molecular Bacteriology and Infection, Department of Life Sciences, Faculty of Natural Sciences, Imperial College London, London, United Kingdom; MRC Centre for Molecular Bacteriology and Infection, Department of Infectious Disease, Faculty of Medicine, Imperial College London, London, United Kingdom; NIHR Health Protection Research Unit in Healthcare Associated Infections and Antimicrobial Resistance, Department of Infectious Disease, Imperial College London, London, United Kingdom; Centre for Antimicrobial Optimisation, Hammersmith Hospital, Imperial College London, London, United Kingdom; Centre for Bio-Inspired Technology, Department of Electrical and Electronic Engineering, Faculty of Engineering, Imperial College London, London, United Kingdom; School of Life Sciences and Department of Statistics, University of Warwick, Coventry, United Kingdom; Department of Mathematics, Imperial College London, London, United Kingdom; NIHR Health Protection Research Unit in Healthcare Associated Infections and Antimicrobial Resistance, Department of Infectious Disease, Imperial College London, London, United Kingdom; Department of Infectious Diseases, Imperial College Healthcare NHS Trust, London, United Kingdom; Centre for Antimicrobial Optimisation, Hammersmith Hospital, Imperial College London, London, United Kingdom; NIHR Health Protection Research Unit in Healthcare Associated Infections and Antimicrobial Resistance, Department of Infectious Disease, Imperial College London, London, United Kingdom; Department of Infectious Disease Epidemiology, School of Public Health, Imperial College London, London, United Kingdom; NIHR Health Protection Research Unit in Healthcare Associated Infections and Antimicrobial Resistance, Department of Infectious Disease, Imperial College London, London, United Kingdom; Department of Infectious Diseases, Imperial College Healthcare NHS Trust, London, United Kingdom; Department of Microbiology, North West London Pathology, London, United Kingdom

**Keywords:** carbapenem-resistant Enterobacterales, IMP carbapenemase, horizontal gene transfer, spatiotemporal network, patient pathways

## Abstract

**Background:**

Carbapenemase-producing Enterobacterales (CPE) are challenging in healthcare, with resistance to multiple classes of antibiotics. This study describes the emergence of imipenemase (IMP)–encoding CPE among diverse Enterobacterales species between 2016 and 2019 across a London regional network.

**Methods:**

We performed a network analysis of patient pathways, using electronic health records, to identify contacts between IMP-encoding CPE–positive patients. Genomes of IMP-encoding CPE isolates were overlaid with patient contacts to imply potential transmission events.

**Results:**

Genomic analysis of 84 Enterobacterales isolates revealed diverse species (predominantly *Klebsiella* spp, *Enterobacter* spp, and *Escherichia coli*); 86% (72 of 84) harbored an IncHI2 plasmid carrying *bla*_IMP_ and colistin resistance gene *mcr-9* (68 of 72). Phylogenetic analysis of IncHI2 plasmids identified 3 lineages showing significant association with patient contacts and movements between 4 hospital sites and across medical specialties, which was missed in initial investigations.

**Conclusions:**

Combined, our patient network and plasmid analyses demonstrate an interspecies, plasmid-mediated outbreak of *bla*_IMP_CPE, which remained unidentified during standard investigations. With DNA sequencing and multimodal data incorporation, the outbreak investigation approach proposed here provides a framework for real-time identification of key factors causing pathogen spread. Plasmid-level outbreak analysis reveals that resistance spread may be wider than suspected, allowing more interventions to stop transmission within hospital networks.

Summary

This was an investigation, using integrated pathway networks and genomics methods, of the emergence of imipenemase-encoding carbapenemase-producing Enterobacterales among diverse Enterobacterales species between 2016 and 2019 in patients across a London regional hospital network, which was missed on routine investigations.

Infections by carbapenemase-producing Enterobacterales (CPE) pose a substantial clinical, operational, and financial challenge [1]. These organisms are associated with high morbidity and mortality rates, and therapeutic options are severely restricted [2]. Carbapenemase genes are frequently carried on plasmids, which can easily transfer between bacterial species [3]. CPE outbreaks involving different bacterial species are often unrecognized, as many plasmids are variable in their gene content and have a broad host range [4]. Outbreaks of Enterobacterales carrying imipenemase (IMP) gene *bla*_IMP-1_ are mostly sporadic and often localized to specific geographic locations [5, 6]. IMP genes are rarely isolated in the United Kingdom, but the number of IMP encoding Enterobacterales species isolates referred to the UK Health Security Agency has been increasing [7].

Colistin and polymyxin B remain the last-line therapeutic agents for CPE in most countries, partly owing to lack of access to newer agents; yet colistin resistance is increasing globally. Ten mobile colistin resistance genes (*mcr-1*—*mcr-10*) have been described to date, presenting a substantial global healthcare challenge [8, 9]. Although *mcr* genes are typically associated with phenotypic polymyxin resistance, *mcr-9* does not appear to confer direct colistin resistance [10, 11] and is widespread in a wide range of bacterial species from human, animal, and environments [11–14].

Person-to-person contact is a route of transmission for many infectious diseases. Consequently, understanding the patterns of these contacts, especially in healthcare settings, can offer detailed insight for targeted interventions [15]. However, such patient contacts become increasingly complex when incorporating multiple layers of data. Network models provide flexible tools to capture complex interactions (contact patterns) and offer a robust and reproducible method that has become widespread across disciplines [16, 17], incorporating both person-to-person transmission through contact networks [18] and spatial spread through networks representing physical locations [19].

So far, few studies have used network models of patient contacts in combination with detailed bacterial genomic analysis and demonstrated the advantages of such an approach by increasing the detail in outbreak characterization [20, 21]. Here, we combine plasmid phylogenomic analysis with patient contact networks to discover the spread of *bla*_IMP_ and *mcr-9* genes among bacterial species and patients in a large hospital network in London, United Kingdom, over 3 years, providing valuable insights for the management of CPE in hospital settings.

## METHODS

### Clinical Setting

This study was carried out using data from a regional network of London hospitals, comprising 7 hospital sites with a total of 2000 inpatient beds, with managerial responsibility assigned to 2 National Health Service trusts, and frequent transfers between trusts and sites for specialist care. Cases were identified from one of these trusts (comprising 5 hospitals), with microbiology and pathway data for those cases identified through a shared centralized microbiology laboratory and electronic health records system (Cerner). Since June 2015, an enhanced routine CPE screening program has been implemented in this trust [22]. When a new case of CPE was identified, the patient was isolated in a single room with contact precautions, the bed space and bathroom were terminally enhanced cleaned, and any contacts were rescreened for CPE.

### Isolate Collection

CPE isolates were collected from patients identified through rectal screens or clinical sampling between June 2016 and November 2019. Bacterial species were determined using Biotyper matrix-assisted laser desorption ionization time-of-flight mass spectrometry (Bruker Daltonics). One isolate per species was collected from each patient. Susceptibility to 21 antimicrobials was tested using the European Committee on Antimicrobial Susceptibility Testing (EUCAST) disc diffusion method, and colistin minimum inhibitory concentrations were retrospectively determined using MICRONAUT broth microdilution (BioConnections) for all viable CPE isolates carrying *bla*_IMP_ genes (hereafter, *bla*_IMP_CPE) [23]. Further phenotypic and molecular characterization of CPE isolates were performed as described in the Supplementary Methods.

### Whole-Genome Sequencing

Isolates of *bla*_IMP_CPE were grown aerobically on Columbia Blood Agar (Oxoid) at 37°C. Genomic DNA was extracted from overnight cultures using GenElute Bacterial Genomic DNA Kits (Sigma-Aldrich). Multiplexed DNA libraries were generated with Nextera XT (Illumina) and sequenced under a 150–base pair paired-end layout for a minimum of 100-fold coverage on Illumina HiSeq 4000 systems (Illumina).

### Phylogenomic Analysis

Quality control of sequencing reads, *de novo* genome assembly, and genetic characterization of isolates are described in the Supplementary Methods. A neighboring-joining tree of CPE genomes was generated from pairwise average nucleotide distances using FastANI software, version 1.33 [24]. Plasmid sequences were reconstructed from genome assemblies using MOB-suite software, version 3.1.0 [25]. Reconstructed sequences of IncHI2 plasmids were aligned against IncHI2 plasmid pKA_P10 (GenBank accession no. CP044215.1), a second isolate from case 71 (IMP42), using Snippy (github.com/tseemann/snippy) to identify genetic variation. A recombination-corrected maximum-likelihood tree of IncHI2 plasmids was reconstructed from the sequence alignment using IQ-Tree software, version 2.0.3 [26], as implemented using Gubbins, version 3.2.1 [27]. The date of the most recent common ancestor of IncHI2 plasmids was estimated using BactDating software, version .1.1.1 [28].

### Network Analysis

To reveal potential transmission structure, a patient contact network was reconstructed from patients’ movement history (ward locations and time), which was extracted from electronic health record data of *bla*_IMP_CPE cases. A contact was defined as an event when 2 patients were present on the same ward on the same day. Time-aggregated patient contacts were subsequently clustered to reveal groups of patients linked together using the Walktrap community detection algorithm [29]. Contacts were weighted by the time spent together, and a temporal analysis of patient interactions was performed to assess patient roles and positions in transmission. A spatial network of ward/hospital distributions was generated, allowing calculation of in-hospital infectious periods—days spent on the ward before the implementation of infection prevention and control (IPC) measures, a network structure to determine ward/hospital spread, and a list of highly visited wards according to plasmid genetic clusters.

To investigate whether the identified lineages of IncHI2 plasmids represented the transmission of *bla*_IMP_CPE, a Kendall rank correlation coefficient was calculated from pairwise phylogenetic distances between IncHI2 plasmids (extracted from the plasmid maximum-likelihood tree) and shortest-path distances between patients (from whom isolates carrying these plasmids were collected) in the contact network (Supplementary Methods).

### Data Availability

Illumina reads and draft genome assemblies of 84 *bla*_IMP_CPE isolates were deposited in European Nucleotide Archive under BioProject PRJEB38818. See Supplementary Table 1 for sample information.

### Ethical Considerations

This study was carried out in accordance with ethics reference 21/LO/0170 (279677; protocol 21HH6538: “Investigation of Epidemiological and Pathogenic Factors Associated With Infectious Diseases”).

## RESULTS

### Incidence of *bla*_IMP_CPE

**Figure 1.**
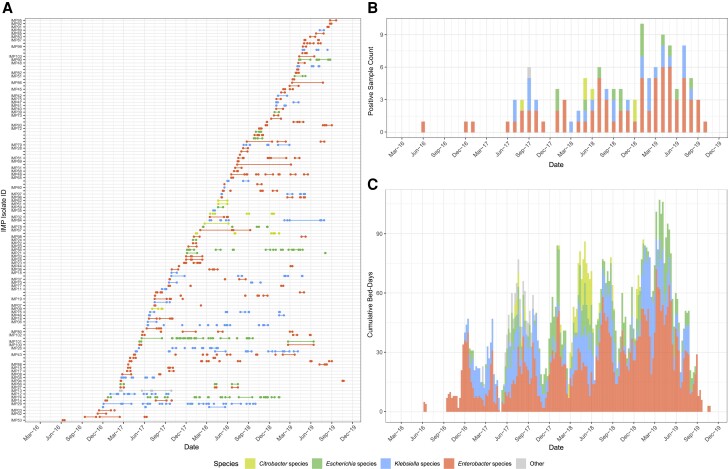
Characteristics of confirmed carbapenemase-producing Enterobacterales (CPE) isolates carrying *bla*_IMP_ genes (*bla*_IMP_CPE) and CPE species. Colors in each panel indicate the genus of CPE. *A*, Total number of bed-days when inpatients (rows labeled by isolate identifier [IMP]) were present in a hospital ward before confirmation of *bla*_IMP_CPE colonization or infection and related infection prevention and control (IPC) measures (in-hospital infectious period). In addition, patients with known carriage of *bla*_IMP_CPE but without sequenced isolates are shown as unlabeled rows. Patients with 2 species of CPE isolates (imipenemase [IMP] 22/24, IMP25/33, IMP96/97, or IMP100/101) are on adjacent rows. *B*, Monthly total number of confirmed *bla*_IMP_CPE isolates from patients during the study period 2016–2019. *C*, Weekly cumulative number of occupied beds in-hospital during the infectious periods. Abbreviations: Mar-16, March 2016 (etc).

Following the introduction of the enhanced CPE screening program, *bla*_IMP_CPE was first observed in 2 trusts’ hospitals in June 2016 through routine rectal screening from a patient with no identifiable travel history. From November 2016, an increasing number of *bla*_IMP_CPE isolates was identified across Enterobacterales species (Figure 1*A*). The highest incidence of *bla*_IMP_CPE cases occurred between January and July 2019 (Figure 1*B*). Altogether, *bla*_IMP_CPE isolates were recovered from screening or clinical samples from 116 patients admitted to these 5 hospitals by the end of November 2019, when numbers of new cases rapidly dropped, and subsequent cases were sporadic and infrequent. No ward or service was identified as a potential focus for cross-transmission, and no enhanced IPC measures were taken, though the overall increase in CPE cases prompted general reinforcement of IPC practices. Only 2 clusters of cases (5 of 116 cases) fit the conventional outbreak definition that ≥2 cases of the same bacterial species with the same resistance mechanism overlapping in time (cases 67 and 71 and cases 80, 81, and 82). Pulsed-field gel electrophoresis typing of CPE isolates showed similar profiles, suggesting within-hospital transmission, and these 2 clusters were dealt with as local CPE transmission events. Furthermore, the daily number of occupied beds revealed a continuous burden of patients colonized with *bla*_IMP_CPE (Figure 1*C*). This burden was particularly evident for patients colonized by *Enterobacter*, with 424 total bed-days in the peak month (March 2019) across the hospital network.

### Contact Network of *bla*_IMP_CPE-Positive Cases

**Figure 2.**
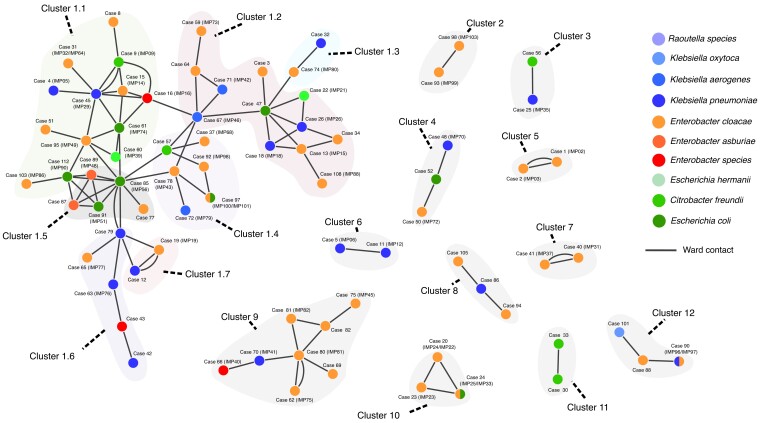
Contact network of carbapenemase-producing Enterobacterales (CPE) isolates carrying *bla*_IMP_ genes (*bla*_IMP_CPE) cases. Each node of the network represents a case, colored according to CPE species (split colors indicate 2 species), and each edge represents a contact between 2 patients, that is, patients present on the same ward on the same day based on their electronic health records. This network contains 12 distinct major clusters (each shaded in light gray, with subclusters 1.1–1.7 shaded in a different color) based on disconnected components of contacts. Cluster 1, the largest cluster, consisting of 45 cases, was further partitioned into 7 subclusters using community detection, with edges weighted by the duration of contact (Supplementary methods—Network community detection). Six patient contacts recurred over different wards, indicated by additional edges connecting the same patients. Abbreviation: IMP, imipenemase.

A detailed patient contact network for 116 *bla*_IMP_CPE cases confirmed that 77 of 116 (66%) were in contact with ≥1 other *bla*_IMP_CPE case (ranging from 1 to 10 cases, with a median of 2; Figure 2 and Supplementary Table 2), creating 96 patient-contact pairs (Supplementary Table 3). Across all contact episodes of *bla*_IMP_CPE cases, 59% of the episodes (57 of 96) involved 2 bla_IMP_CPE species and were therefore excluded from the conventional same-species definition of an outbreak when initially reviewed. The contact network split patients into 12 separate clusters, with interactions occurring across different hospitals, as patients were transferred between wards and hospital sites (Figure 2). The largest contact cluster (cluster 1) contained 45 patients and was further partitioned into 7 subclusters (labeled from 1.1 to 1.7) that comprised 13, 12, 2, 6, 5, 5, and 2 patients, respectively (Figure 2). The analysis of contacts at regional, hospital, and ward levels suggested involvement of different *bla*_IMP_CPE species in patient-to-patient transmission events and prompted phylogenomic analysis of available *bla*_IMP_CPE isolates.

### Genomic and Phenotypic Characterization of *bla*_IMP_CPE Isolates

**Figure 3.**
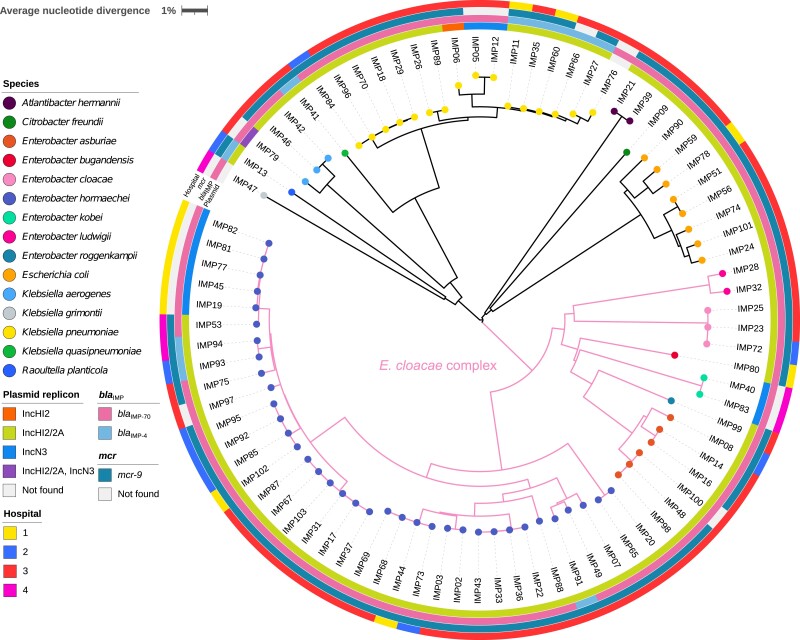
A neighbor-joining tree of 84 carbapenemase-producing Enterobacterales isolates carrying *bla*_IMP_ genes. This tree was constructed from average nucleotide distances between genomic sequences and was midpoint rooted. The color at the end of each branch indicates the bacterial species identity for that isolate. The innermost ring indicates the type of plasmid detected; the second ring, the allelic variant of the *bla*_IMP_ gene detected; the third ring, the presence or absence of gene *mcr-9*; and the outermost ring, hospitals. The scale bar indicates the pairwise average nucleotide divergence (as a percentage). Abbreviation: IMP, imipenemase.

A total of 84 *bla_IMP_*CPE isolates (collected from 82 of 116 case patients) were available and viable for whole-genome sequencing (WGS; Supplementary Table 1). These isolates belonged to 15 species and were dominated by those of the *Enterobacter cloacae* complex (n = 51), followed by *Klebsiella* spp (n = 21) and *Escherichia coli* (n = 8) (Figure 3). Four patients (patients 20, 24, 90, and 97) were colonized by 2 *bla*_IMP_CPE species (Supplementary Table 2).

Each of these 84 isolates carried either *bla*_IMP-70_ (n = 74) and *bla*_IMP-4_ (n = 10). IncHI2 plasmids (targeted by both probes for IncHI2 and IncHI2A replicons in the PlasmidFinder database) were detected in 72 isolates from 72 cases, and IncN3 plasmids were detected in 10 isolates from 10 cases (Figure 3). These plasmids were predicted to be conjugative with MOB-suite software for the presence of genes encoding MOB_H_-family relaxases, type-F mating pair formation, and the origin of transfer (*oriT*). We have identified *intI1* genes, and aminoglycoside resistance gene *aac(6′)-Ib3* was found downstream of *bla*_IMP_, implying presence of the known multidrug-resistant class 1 integron harboring *bla*_IMP_ in IncHI2 plasmids [30].

Only IMP79 harbored both IncHI2 (without any *bla*_IMP_ genes) and IncN3 (carried *bla*_IMP-70_) plasmids. Seventy of the 72 IncHI2 plasmids carried either *bla*_IMP-70_ (n = 61) or *bla*_IMP-4_ (n = 9), and all IncN3 plasmids carried *bla*_IMP-70_ (Supplementary Tables 4 and 5). Four isolates carried *bla*_IMP_ genes that were not found in either IncHI2 or IncN3 plasmids: 1 *bla*_IMP-4_ was integrated into the chromosome of IMP66, and *bla*_IMP-70_ was carried by an IncFIB/FII plasmid in IMP83 and by IncHI1 plasmids in IMP47 and IMP76. All *bla*_IMP_CPE isolates carried multiple β-lactam resistance genes and other antimicrobial resistance genes, yet only IMP89 had an additional carbapenem-resistance gene *bla*_OXA-48_ (Supplementary Table 1).

Gene *mcr-*9 was detected in 69 of 84 (82%) isolates, with *mcr-9* identified present on 68 IncHI2 plasmids, 1 outlier IncHI2 plasmid (32% coverage of the 334–kilobase pair reference plasmid pKA_P10 by sequencing reads), and none of the IncN3 plasmids (Supplementary Tables 4 and 5). The *mcr*-9 LAMP (Loop-mediated isothermal amplification) assay showed 100% concordance with the WGS results. MALDIxin testing did not reveal any lipid A modifications attributable to the *mcr*-9 gene in this study. Altogether, 12 isolates (all *Enterobacter*) were resistant to colistin (minimum inhibitory concentrations ranged between 4 and >64 μg/mL), including 5 that demonstrated a skipped-well phenomenon suggestive of colistin heteroresistance (Supplementary Table 1), a phenomenon reported elsewhere [31].

### Genetic Relatedness Between Plasmids

All 72 reconstructed IncHI2 plasmids belonged to the same plasmid taxonomic unit PTU-HI2 and overlapped with 65%–89% of the reference sequence pKA_P10. Representative sequences of these plasmids are compared in Supplementary Figure 1. Altogether, 144 single-nucleotide polymorphic sites were identified in the alignment of these 72 plasmids after correcting for recombination events, with pairwise phylogenetic distances (sums of branch lengths in the plasmid tree) ranged from 0 to 115 single-nucleotide polymorphisms (SNPs). Specifically, of the 72 plasmids analyzed in the tree, 43 (60%) differed by ≤3 SNPs, and 55 (76%) differed by ≤5 SNPs. This high degree of similarity between IncHI2 plasmids suggests potential horizontal gene transfer or transfer of full plasmids between different bacterial species. In the case of IncHI2 plasmids present in the same species, a comparison between the plasmid and *Enterobacter hormaechei* (the most common species in our data) phylogenetic trees showed likely vertical transmission events as closely related isolates had highly similar plasmids (Supplementary Figure 2). By contrast, reconstructed IncN3 plasmids showed large structural variation in the plasmids (Supplementary Figure 3), and no reliable phylogenetic tree could be reconstructed.

**Figure 4.**
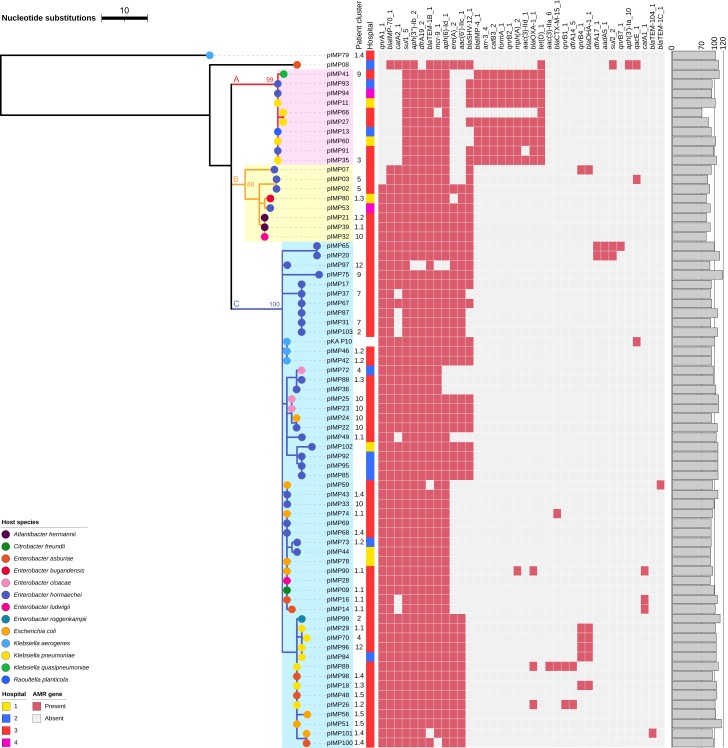
Recombination-corrected maximum-likelihood tree of 72 reconstructed IncHI2 plasmids from carbapenemase-producing Enterobacterales (CPE) isolates carrying *bla*_IMP_ genes (*bla*_IMP_CPE) and the reference plasmid pKA_P10. Colored branches and shades represent plasmid lineages A, B, and C, with bootstrap values of lineage roots noted. Heat map shows the presence or absence of antimicrobial resistance genes identified in plasmids, and the bar plot shows relative lengths (as percentages) of the reconstructed plasmids compared with the reference plasmid pKA_P10. This tree is rooted on the outgroup pIMP79, which was deemed an outgroup according to its phylogenetic distances to other IncHI2 plasmids and by the BactDating root-to-tip analysis (Supplementary Figure 4). Abbreviation: IMP, imipenemase.

The phylogenetic tree of IncHI2 plasmids indicated 3 major lineages A, B, and C (Figure 4 and Supplementary Table 6). All IncHI2 plasmids from the 4 cases of dual-species bla_IMP_CPE colonization belonged to lineage C. The estimated date of the most recent common ancestor of the 72 IncHI2 plasmids was 1765, with a large 95% confidence interval of 1536–1895, despite a desirable convergence of the optimized molecular clock model (Supplementary Figure 4), suggesting a lack of temporal signals in reconstructed IncHI2 plasmids.

### Comparison Between Plasmid Lineages and Patient Clusters

Pairwise phylogenetic distances between IncHI2 plasmids and shortest-path contact distances between patients showed a significant correlation (Kendall correlation coefficient = 0.19; *p* = 0.0000003), despite WGS data being unavailable for isolates from 24 cases. This correlation between plasmid population structure and patient contact network suggests that ward contacts mediated transmission of these plasmids between patients or from unidentified common sources. When case contacts were weighted by patients’ time spent together, the *bla*_IMP_CPE outbreak was heavily weighted toward hospital 3, the specialist referral center for cardiology, renal, hematology, and hepatobiliary services, with 72.1% of contacts occurring there.

**Figure 5.**
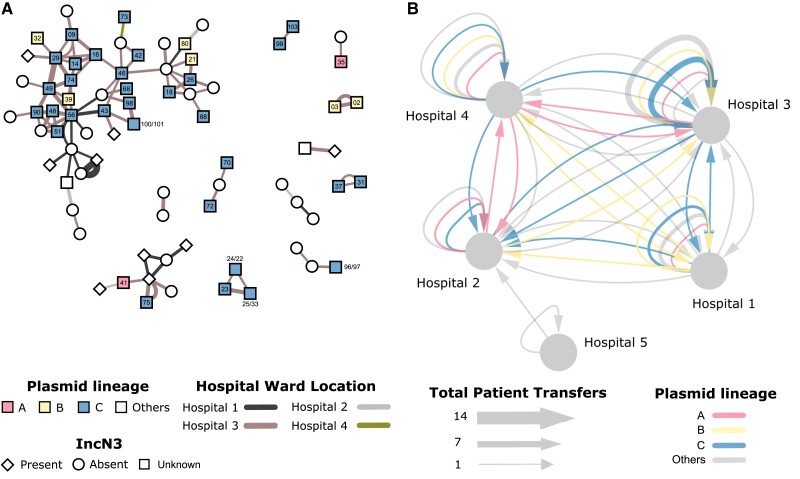
Epidemiology of *bla*_IMP_CPE genetic clusters across patient interactions and movement. (A) Patient contact network overlaid with plasmid lineages A, B, and C. Each node represents a patient, edges represent recorded ward contacts between confirmed *bla*IMPCPE cases. The edges are coloured according to the hospital site and the width of the edge is proportional to the duration of the contact (143 days). Nodes are coloured according to the three lineages of IncHI2 plasmids, and patients with isolates that did not have any IncHI2 plasmids detected are uncoloured. Node labels indicate IncHI2 plasmid names in Supplementary Table 2 (eg, ‘88’ indicates plasmid pIMP88, and ‘100/101’ indicates plasmids pIMP100 and pIMP101 from the same case). The presence/absence of IncN3 plasmids in *bla*IMPCPE isolates is denoted by node shapes. (B) Hospital-level patient movements. The movement of patients carrying *bla*IMPCPE are indicated by arrows between hospitals. Repeated transfers of patients between wards are aggregated into edges with proportionally greater edge widths (grouped by sequenced and non-sequenced). Edges with sequencing data are coloured according to IncHI2 plasmid lineages. This network was generated from Supplementary Table 3 and visualized using Cytoscape software, version 3.10.1 [32]. Abbreviation: IMP, imipenemase.

The role of hospital 3 as the center of this outbreak was confirmed by the analysis of the spatial distribution and movement of patients colonized with *bla*_IMP_CPE carrying IncHI2 plasmids (Figure 5). The largest lineage (lineage C) was found to be the most prevalent on wards within hospital 3 (1919 patient bed-days) and followed bidirectional transfer pathways to and from hospitals 1, 2, and 4, which all have large general medical and surgical admissions areas. Lineage A followed a similar pattern of distribution, though with fewer transfers identified to hospital 2, which may have been due to unidentified or missing case data.

The association between plasmid lineages and ward/specialties over the study period showed the most common associations across critical care and renal services (Supplementary Table 7). The only exception was general internal medicine and general surgery predominated in plasmid lineage A at hospital 4, which has more general wards and less specialist services than the other hospital sites in the network. Despite the predominance of cases being identified in specialties with high risk for invasive disease, only 4 clinical infections were identified during the study, and no bloodstream infections.

## DISCUSSION

Following the detection of a new mechanism of resistance, investigation of its origin and mode of transmission is challenging, especially in healthcare settings, where investigations usually focus on single-species transmissions. With confounding factors such as multiple bacterial species and spread over different hospital locations, new methods to investigate potential outbreaks are much needed. The incorporation of plasmid genomics and patient networks into our analysis changed the way the emergence of *bla*_IMP_CPE was visualized and produced a clearer understanding of the cumulative burden of cases, high-risk ward locations, and pathways for potential cross-transmission in our regional healthcare system. As patients were found to follow common routes, with regular reencounters, this information can provide dynamic risk assessments to be introduced along those pathways, to prevent future cross-transmission events of any healthcare-associated pathogen [33]. Detailed genomic analysis of plasmids enhanced our understanding of the relatedness of different patient isolates to the network analysis and of the similarity to those plasmids identified in other hospitals in the United Kingdom [30]. It moreover revealed concerning information about unsuspected resistance mechanisms, with potential for antibiotic treatment failures that were missed by conventional laboratory susceptibility testing.

In the current study, we characterized IncHI2 plasmids as the main vehicle in horizontal transfer of the metallo-β-lactamase gene *bla*_IMP_ and colistin resistance gene *mcr-9*. These 72 plasmids were predominant in multiple bacterial species across epidemiologically linked patients, highlighting the need for integration of genomics into routine clinical practice. The *mcr-9–*carrying IncHI2 plasmids have been identified from human, animal, and environmental samples globally [12, 14]. These plasmids are known to carry integron-associated genes encoding resistance against aminoglycosides, β-lactams (eg, by *bla*_CTX-M_, *bla*_IMP_, *bla*_VIM_, and *bla*_NDM_ genes), chloramphenicol, macrolides, quinolones, rifamycin, sulfonamides, tetracycline, and trimethoprim, as well as heavy-metal resistance genes, and they facilitate the transmission of these between bacterial species [12, 14, 34–36]. Notably, we did not find phenotypic expression of *mcr-9* although we detected this gene in 69 of the 84 isolates (82%) in our study, which is in line with previous reports [11, 37]. Bacterial hosts have been shown to maintain IncHI2 plasmids long term, even when exposed to different conditions (presence or absence of antimicrobials or nutrient-rich or nutrient-restricted culture medium); this has been attributed to the plasmid–chromosome coevolution that helps reduce fitness costs of the plasmid while compromising its conjugation capacity [38, 39]. This may have contributed to the successful proliferation of IncHI2 plasmids in our hospital network.

Our study supports the concept that plasmid analysis across different resistance mechanisms, as well as among different species, should be the standard for investigations in the future. Network analyses and cumulative burden analyses can help identify targets for WGS, particularly where resources are not sufficient to support WGS of all new CPE cases identified. The small number of clinical infections from this outbreak compared with other CPE outbreaks from our hospital network [40] and other reports of *bla*_IMP_CPE [11, 14] is noteworthy, and it poses questions about the wider importance of this plasmid and the resistance mechanisms revealed in this study. This observation reinforces the argument that screening for silent carriage of CPE in hospitals is key to preventing spread [41–43], and cautious antimicrobial stewardship is essential to prevent expression of hidden resistance mechanisms [44].

We acknowledge several limitations of our study. First, we did not have long-read sequences to recover complete plasmid sequences. As a result, our plasmid tree may omit some similarities and differences between identified IncHI2 plasmids. Furthermore, a comparison between complete sequences of IncHI2 plasmids from our hospital network and those from UK or global collections is not presented here and is the subject of future work. Second, full pathway data across the hospital during the 3 years of the outbreak were available only for identified positive cases, not for all patients in the hospitals during the study period. It was therefore not possible to fully establish potential missed cases flagging as close contacts but with potential for missed screening or false-negative results. Full pathway movement data for all positive cases identified within our hospital network were available, yet neither pathway details nor genomic data were available for other *bla*_IMP_CPE-positive patients identified in the 2 other regional hospitals who did not visit our institution, thus reducing the understanding in our analysis. Third, interactions at other potential hospital locations such as interventional imaging or endoscopy were not examined in this study, nor was environmental sampling performed, which could inform future studies on modes of transmission.

Nevertheless, this study highlights a previously unidentified extent of transmission and thus provides valuable new insights into the spread of an emerging resistance mechanism. Moreover, our novel multilayered methods, incorporating plasmid phylogeny with contact network analysis, provide invaluable tools for outbreak investigation that can be generalized to a wide range of scenarios.

## Supplementary Data

Supplementary materials are available at *The Journal of Infectious Diseases* online (http://jid.oxfordjournals.org/). Supplementary materials consist of data provided by the author that are published to benefit the reader. The posted materials are not copyedited. The contents of all supplementary data are the sole responsibility of the authors. Questions or messages regarding errors should be addressed to the author.

## Supplementary Material

jiae019_Supplementary_Data

## Notes

***Acknowledgments.*** We thank the staff of the diagnostic microbiology laboratory of North West London Pathology for isolate collection and storage. We also acknowledge the support of the Imperial College Healthcare Trust National Institute for Health Research (NIHR) Biomedical Research Centre. The Imperial Biomedical Research Centre's Genomics Facility has provided resources and support that have contributed to the research results reported within this article; it is supported by NIHR funding to the Imperial Biomedical Research Centre. This publication made use of the PubMLST website (https://pubmlst.org/), developed by Jolley and Maiden [45, 46] and sited at the University of Oxford; the development of the website was funded by the Wellcome Trust.

***Author contributions.*** Conceptualization: F. B., M. J. E., X. D., M. B., A. H. H., E. J., and F. D. Data curation: Y. W., A. C. M., S. M., A. Y. W., M. J. E., E. J., F. D., and A. B. Formal analysis: Y. W., A. C. M., F. B., A. Y. W., M. G., M. J. E., M. B., A. H. H., E. J., and F. D. Funding acquisition: A. B., M. J. E., A. H. H., and F. D. Investigation: Y. W., A. C. M., A. B., A. L., J. F. T., H. A., R. P., A. S., A. A., K. M. C., L. M., G. L. M., and J. R. M. Methodology: A. C. M., S. M., A. Y. W., F. D., Y. W., M. G., J. F. T., K. L. H., M. J. E., J. A. O., X. D., M. B., E. J., and A. B. Project administration: F. B., A. H. H., E. J., and F. D. Resources: H. D., K. L. H., A. M. E., A. H. H., and F. D. Supervision: M. B., A. H. H., E. J., and F. D. Validation: S. M., A. Y. W., J. F. T., H. D., K. L. H., and M. J. E. Visualization: Y. W., A. C. M., A. B., E. J., and F. D. Writing—original draft and further drafts: Y. W., A. C. M., A. B., F. B., E. J., and F. D. Writing—review and editing: All authors.

***Disclaimer.*** The views expressed in this publication are those of the authors and not necessarily those of the National Health Service, the National Institute for Health Research, the Department of Health and Social Care, or the UK Health Security Agency.

***Financial support*.** This work was funded in part by the Faculty of Medicine, Siriraj Hospital, Mahidol University, Thailand (awarded to A. B.) and Medical Research Council (Clinical Academic Research Fellowship scheme, MR/T005254/1, awarded to F. D.). The authors also acknowledge funding from the Medical Research Foundation (National PhD Training Programme in Antimicrobial Resistance Research, MRF-145-0004-TPG-AVISO, to A. C. M.); the EPSRC (grant EP/N014529/1 to M.B.) supporting the EPSRC Centre for Mathematics of Precision Healthcare at Imperial College London; The Medical Research Council (Doctoral Training Award to Imperial College London, MR/N014103/1, to A. S.); Rosetrees Trust and the Stoneygate Trust (Imperial College Research Fellowship, M683, to E. J.); Wellcome Trust and Imperial College London (Institutional Strategic Support Fund Springboard Research Fellowship, PSN109, to Y. W.); the Imperial Health Charity (to H. A.); The NIHR (Imperial Biomedical Research Centre at Imperial College London; the Health Protection Research Unit in Genomics and Enabling Data at Warwick University to X. D.; Senior Investigator award to A. H. H.; Health Protection Research Unit in Healthcare Associated Infections and Antimicrobial Resistance at Imperial College London, NIHR200876, to A. H. H.). A.L. is affiliated with the National Institute for Health Research Health Protection Research Unit (NIHR HPRU) in Modelling Methodology at Imperial College London (grant HPRU-2012–10080) and the National Institute for Health Research Health Protection Research Unit in Healthcare Associated Infections and Antimicrobial Resistance at University of Oxford (NIHR200915) in partnership with UK Health Security Agency. A. H. H., F. D., E. J., A. B., Y. W., M. J. E., M. G. are affiliated with the National Institute for Health Research Health Protection Research Unit (NIHR HPRU) in Healthcare Associated Infections and Antimicrobial Resistance at Imperial College London in partnership with the UK Health Security Agency (previously PHE), in collaboration with, Imperial Healthcare Partners, University of Cambridge and University of Warwick. This report is independent research funded by the National Institute for Health Research.
