# Supplementary material for: Integrated Analysis of Patient Networks and Plasmid Genomes to Investigate a Regional, Multispecies Outbreak of Carbapenemase-Producing Enterobacterales Carrying Both blaIMP and mcr-9 Genes
Source: J Infect Dis. 2024 Jan 20;230(1):e159–70. doi: 10.1093/infdis/jiae019 (PMC11272044; doi:10.1093/infdis/jiae019)
Supplement: jiae019_Supplementary_Data [file jiae019_supplementary_data.zip › 20231222_supplmentary_figures.docx]

**Integrated patient network and genomic plasmid analysis reveals a regional, multi-species outbreak of carbapenemase-producing Enterobacterales carrying both *bla*_IMP_ and *mcr-9* genes**

Supplementary Figures


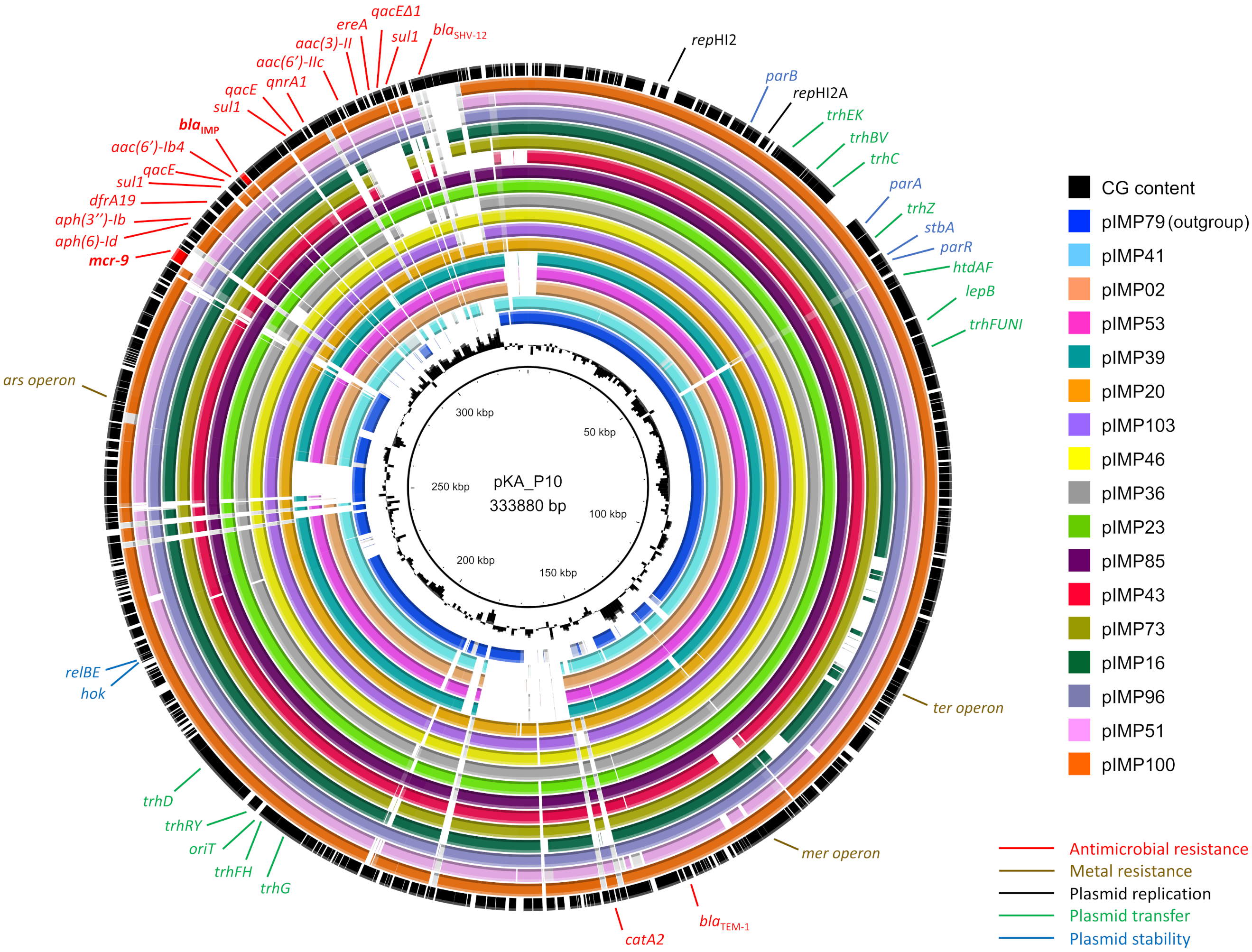


Supplementary Figure 1. BRIG diagram comparing MOB-suite-reconstructed IncHI2 plasmids to reference sequence pKA_P10 (CP044215.1). Sixteen representative plasmids were selected from the phylogenetic tree. The reconstructed outgroup plasmid pIMP79 is also included for comparison. The innermost ring (black) represents the GC content, and the outermost ring (black) shows genes in the reference. Each ring of other colours represents homologous regions shared between a specific reconstructed plasmid and the reference based on ≥90% (coloured according to the legend) and between 70–90% (grey) nucleotide identities. Genes *bla_IMP_* (pIMP41: *bla*_IMP-4_; other 15 representatives: *bla*_IMP-70_; pIMP79: none) and *mcr-9* in the reference sequence are highlighted in red in the outer-most ring.


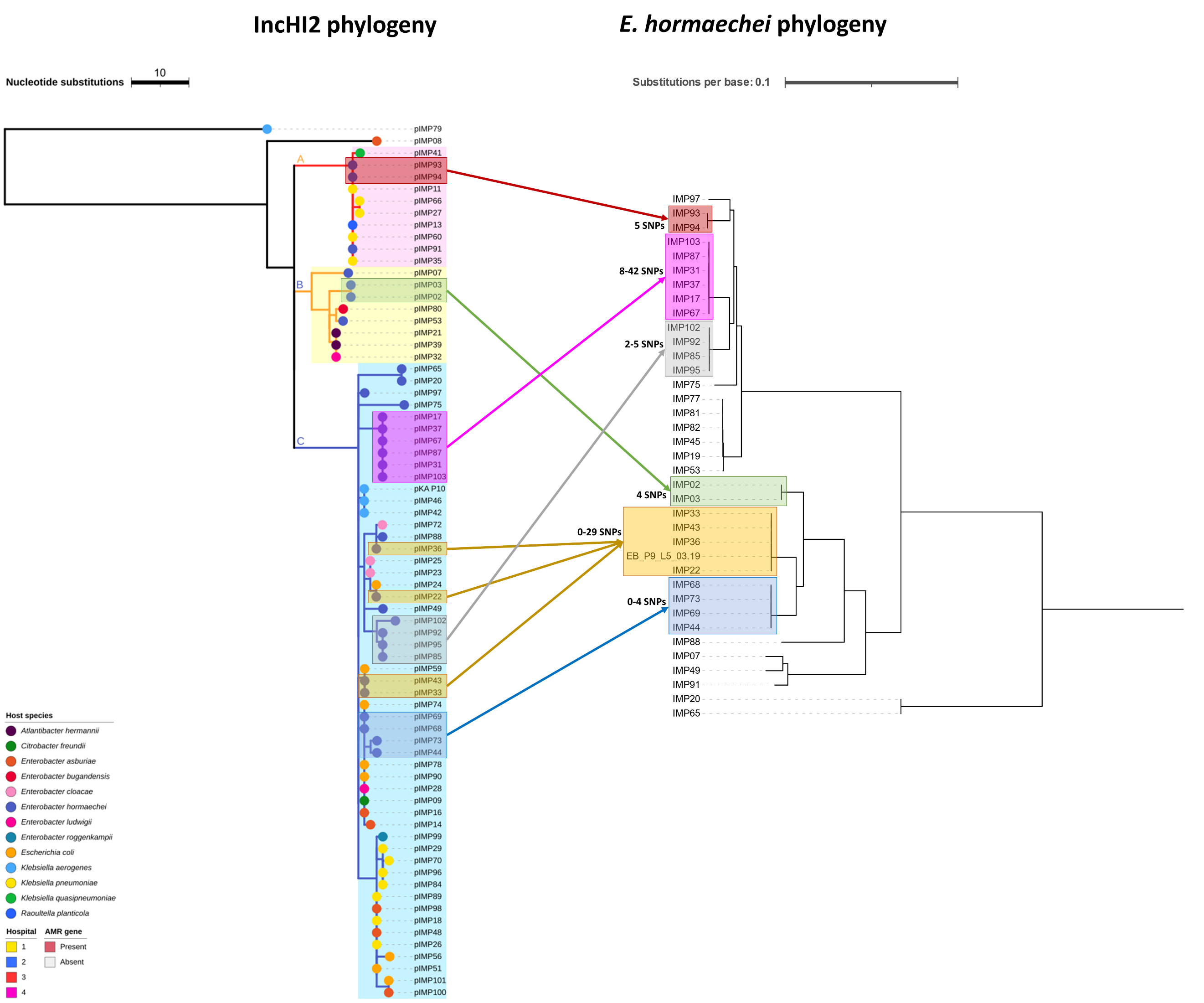


**Supplementary Figure 2**. **Linking IncHI2 plasmids to host *E. hormaechei* isolates**. Left: the same phylogenetic tree of IncHI2 plasmids as that in Figure 4 in the main text, where tip circles (representing plasmids) are coloured by host bacterial species (*E. hormaechei*: dark blue) and three plasmid lineages (A, B, C) are labelled and indicated with distinct colours. Right: a midpoint-rooted maximum-likelihood phylogenetic tree of *E. hormaechei* isolates generated through mapping Illumina reads against the reference chromosome sequence of *E. hormaechei* isolate EB_P9_L5_03.19 (GenBank accession: CP043766.1) with Snippy and recombination-corrected phylogenetic reconstruction with IQ-Tree as implemented in Gubbins. *E. hormaechei* isolates carrying IncHI2 plasmids were clustered based on a maximum of 50 single-nucleotide substitutions (SNPs) or nucleotide substitutions, with each cluster highlighted by a colour shade and noted by the range of pairwise genetic divergence (measured by SNPs) within the cluster.

**
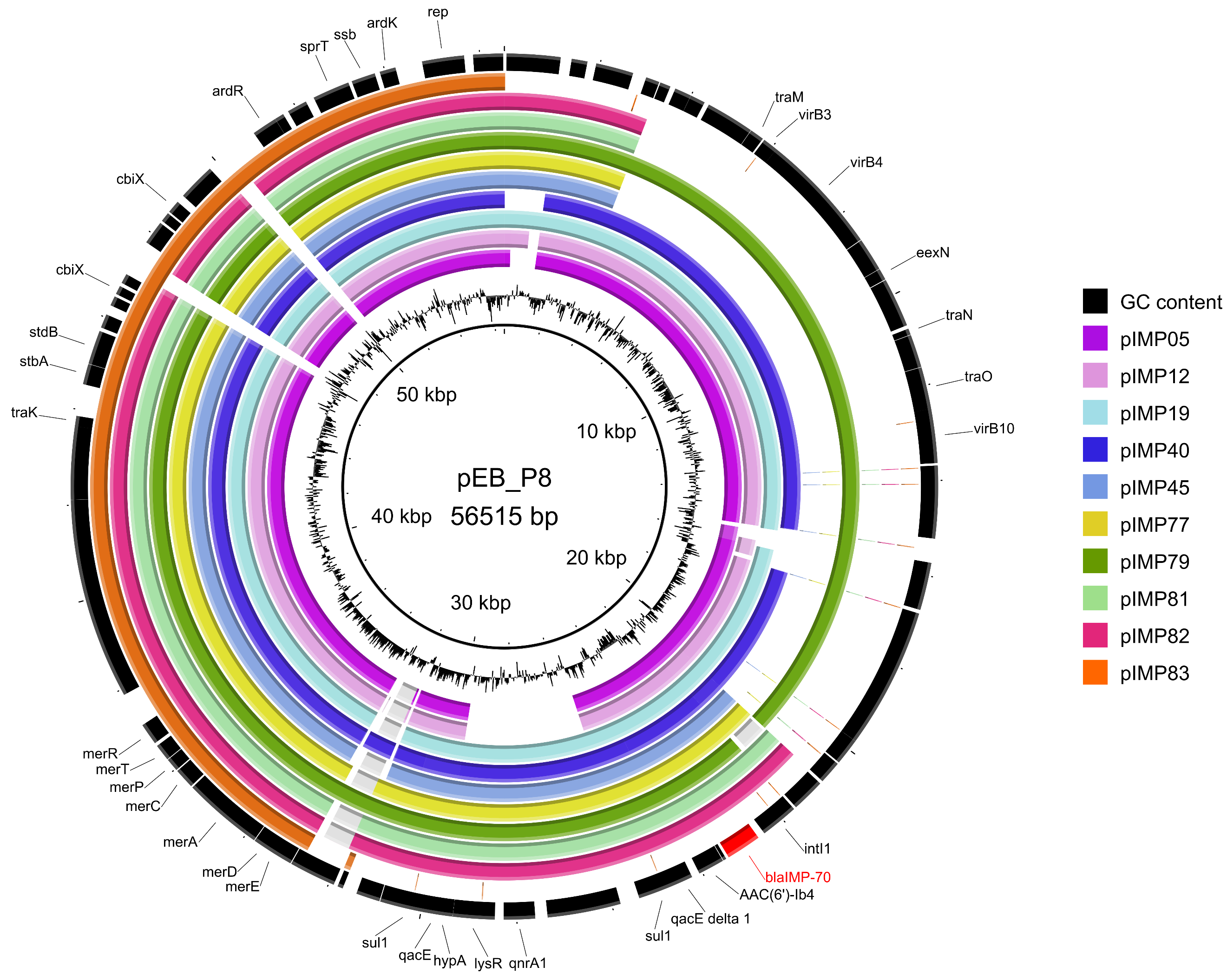
**

**Supplementary Figure 3. BRIG diagram comparing MOBSuite-reconstructed IncN3 plasmids to reference sequence pEB_P8 (GenBank accession: CP043516.1).** The innermost ring (black) represents the GC content, and the outermost ring (black) shows genes in the reference. Gene *bla_IMP-70_* is highlighted in red in the outermost ring. Each ring of other colours represents homologous regions shared between a specific reconstructed plasmid and the reference based on ≥90% (coloured according to the legend) and between 70–90% (grey) nucleotide identities.

**
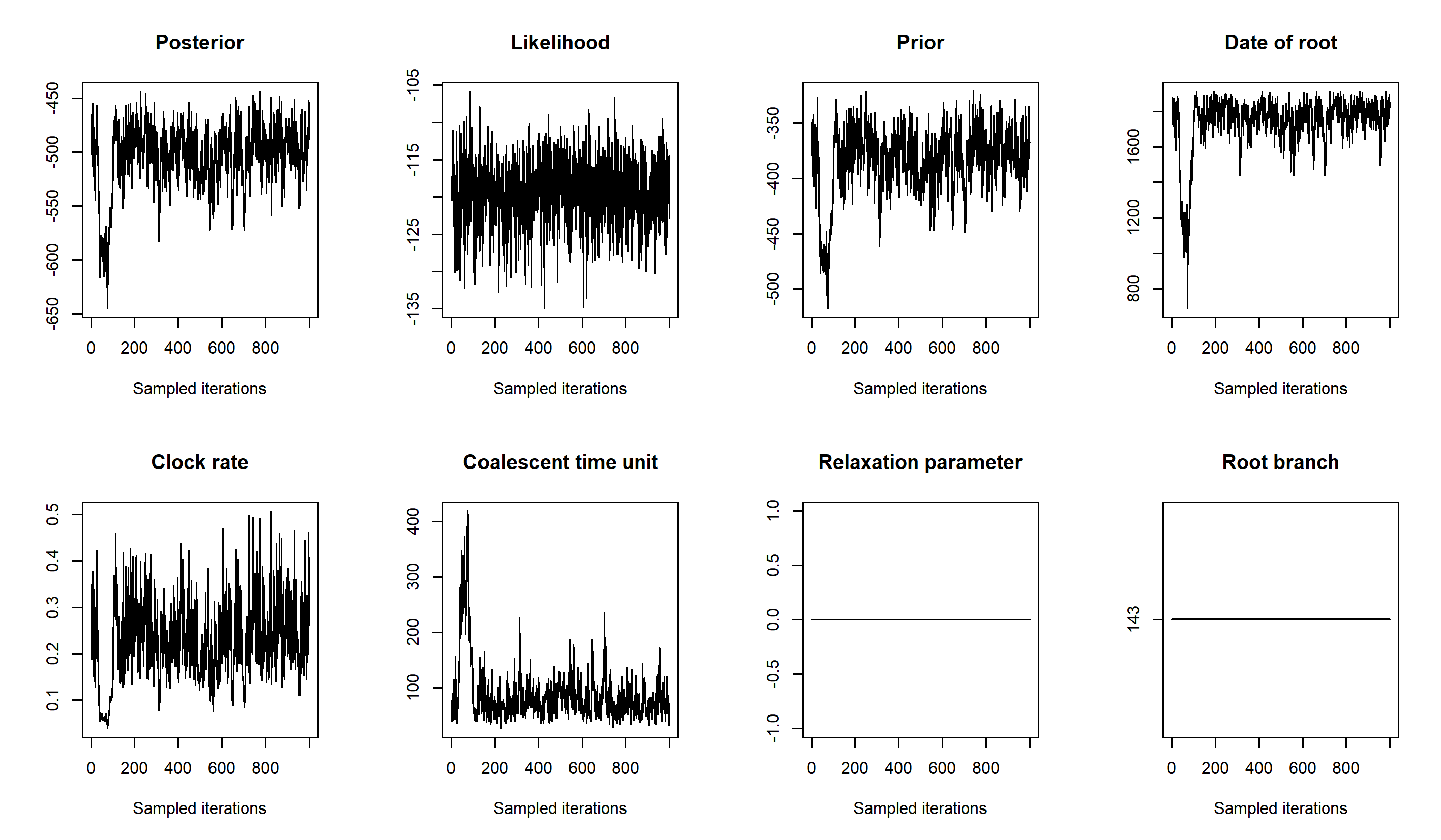
**
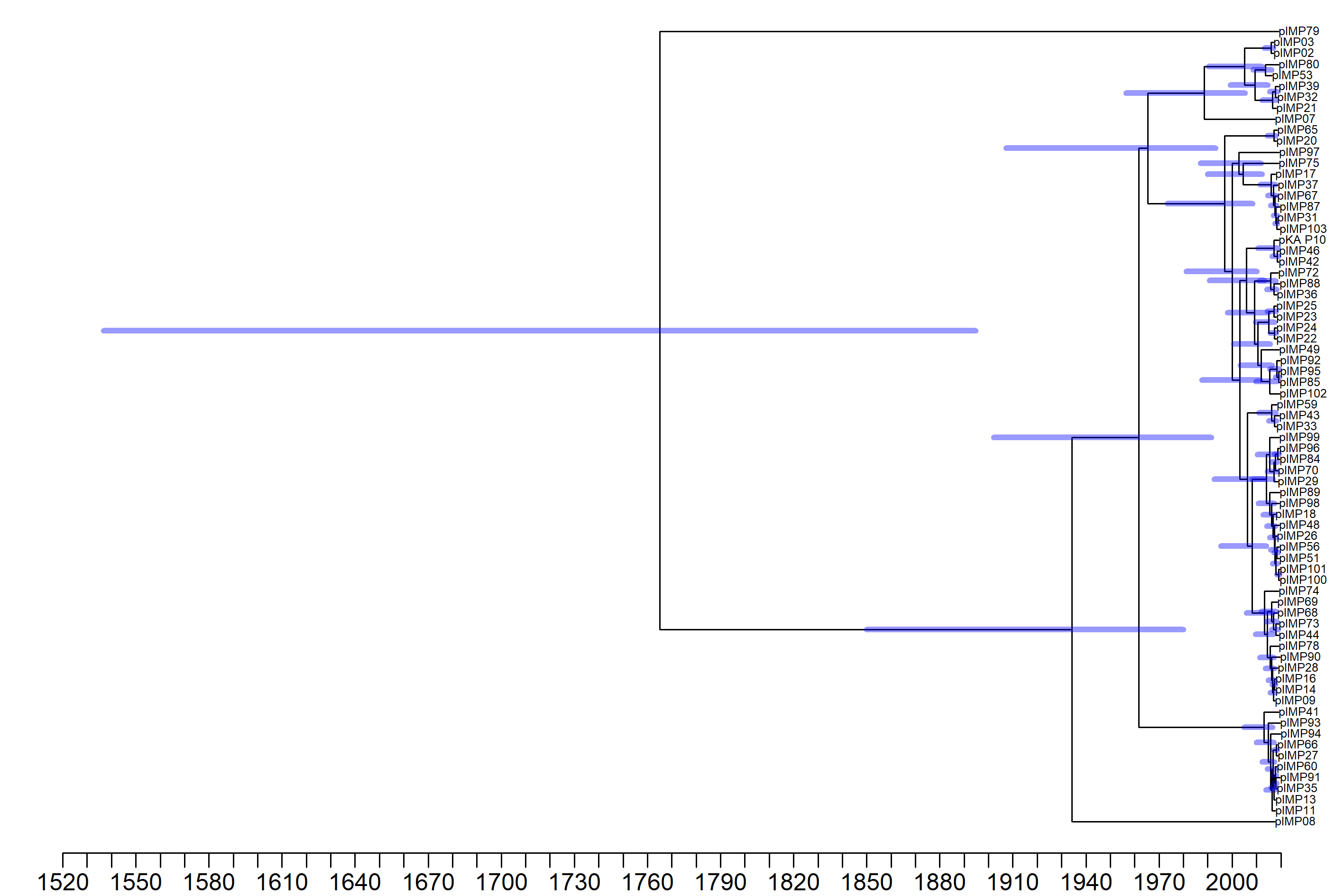


**(b)**

**(a)**

**Supplementary Figure 4. Estimated ancestral dates of 73 IncHI2 plasmids**. **(a)** Blue bars indicate 95% CI of mean dates when common ancestors of corresponding branches existed by estimation. The root of the tree was inferred by the root-to-tip algorithm executed by function *bactdate* (parameter *updateRoot = TRUE*) of package BactDating. **(b)** Parameter convergence as shown by traces of parameter estimates from 2×10^6^ iterations of Markov Chain Monte Carlo. A strict molecular clock, as specified by the Poisson model for BactDating analysis, was determined as the best model for estimating evolutionary rates and ancestral dates of the 73 IncHI2 plasmids (72 plasmids from this study and reference plasmid pKA_P10) when taking recombination events into account. The most recent common ancestor of all these plasmids was dated to 1765 (95% confidence interval [CI]: 1536–1895). Nevertheless, estimated ancestral dates showed large and overlapping 95% CIs throughout the plasmid tree despite a desirable parameter convergence, suggesting a lack of temporal signals in reconstructed IncHI2 plasmids even though recombination events have been considered.
