# Supplementary material for: Integrated Analysis of Patient Networks and Plasmid Genomes to Investigate a Regional, Multispecies Outbreak of Carbapenemase-Producing Enterobacterales Carrying Both blaIMP and mcr-9 Genes
Source: J Infect Dis. 2024 Jan 20;230(1):e159–70. doi: 10.1093/infdis/jiae019 (PMC11272044; doi:10.1093/infdis/jiae019)
Supplement: jiae019_Supplementary_Data [file jiae019_supplementary_data.zip › 20240102_supplementary_methods.docx]

**Integrated patient network and genomic plasmid analysis reveals a regional, multi-species outbreak of carbapenemase-producing Enterobacterales carrying both *bla*_IMP_ and *mcr-9* genes**

Supplementary Methods

# Detection of carbapenemases and carbapenemase genes

Prior to February 2018, Enterobacterales isolates were tested for resistance against ertapenem/meropenem/imipenem using various assays in the two Trusts’ central microbiology laboratories. Carbapenemase genes in resistant isolates were detected with the PCR-based Xpert Carba-R assay (Cepheid Inc, USA). Resistant isolates with negative Xpert Carba-R results were sent to the national reference laboratory at UK Health Security Agency (UKHSA, formerly Public Health England) for confirmation of resistance mechanisms.

From February 2018, as previously described [1], all carbapenem-resistant isolates were tested for carbapenemase production and identification using the RESIST-3 O.K.N. K-SeT kit (Coris BioConcept, Belgium) in the central microbiology laboratories. Carbapenemase-negative isolates were retested using the carbapenem inactivation method (CIM) [2], and carbapenemase genes in carbapenemase/CIM-positive isolates were detected with the Xpert Carba-R assay. Moreover, isolates carrying *bla*_IMP_ and having suspected patient links were sent to UKHSA for molecular typing (Variable Number Tandem Repeat for *Klebsiella* and Pulsed Field Gel Electrophoresis for other species).

# Detection and characterisation of colistin resistance

Loop-mediated isothermal amplification (LAMP) was used to examine the prevalence of *mcr-9* in *bla*_IMP_CPE isolates [3]. In addition to MICRONAUT broth microdilution assay for colistin susceptibility of isolates, MALDIxin testing [4], which detects modifications to the lipid A of the lipopolysaccharide as mediated by *mcr*1-5 and *mcr*-8, was conducted on isolates IMP1–IMP58 to further characterise colistin susceptibility.

# Phylogenomic analysis

Quality assessment of raw sequencing reads was performed with FastQC v0.11.6 and low quality reads were removed using Trimommatic v0.39 [5,6]. Bacterial species were verified using Kraken v2.1.2, Bracken v2.6.2, and KmerFinder v1.4.3 [7–9]. Genome assemblies of CPE isolates were generated from trimmed reads using Unicycler v0.5.0 [10]. Multi-locus sequence typing, identification of plasmid replicons and acquired AMR genes were carried out using ABRicate v1.0.1 and databases of PubMLST, BIGSdb-Pasteur, ResFinder, and PlasmidFinder [11–14].

MOB-suite v3.1.0 [15] was used to reconstruct plasmids from genome assemblies. First, program MOB-init was used to download the corresponding databases. Then, two reference sequences of IncHI2 plasmids, pKA_P10 (NZ_CP044215.1) and pEB_P9 (NZ_CP043767.1), were added to the plasmid database using MOB-cluster. Finally, MOB-recon was used to identify and reconstruct plasmids based on the new database, using 500 bp as the minimum length of contigs included. Reconstructed sequences of IncHI2 plasmids were analysed with COPLA v1.0 to identify plasmid taxonomic units [16]. Plasmid comparison and visualisation were performed with BLAST Ring Image Generator v0.95 [17], using pKA_P10 as the reference for IncHI2 plasmids and pEB_P8 (CP043516.1) as the reference for IncN3 plasmids.

Function *modelcompare* in the Bactdating v.1.1.1 package [18] was used to select the best molecular clock model in the phylogenetic dating analysis of IncHI2 plasmids. The same methods for sequence alignment and phylogenetic reconstruction were applied to reconstructed sequences of IncN3 plasmids with plasmid pEB_P8 (GenBank accession: CP043516.1) as the reference. However, the recombination-corrected maximum-likelihood tree of IncN3 plasmids was not informative because bootstrap values were generally low (2–35%).

# Construction of contact networks

Ward locations and time were extracted from the Electronic Health Record data for *bla*_IMP_CPE cases to establish patient-movement history. Movement to other locations for medical procedures during an inpatient episode, such as endoscopy, interventional radiology or operating theatres, was not examined.

We considered the hospital movement of $N$ patients under investigation represented by a set of pathways $\varphi=\{P_{1}, P_{2},..., P_{N}\}$. Each patient pathway $P_{N}$ consists of an ordered set of ward-timings pairs $P_{N}=\{l_{1},l_{1},...,l_{k_{n}}\},$ where $k_{n}$ is the number of location-timings for individual $n$, and each ward-timing $l_{i} = (v_{i}$,$t_{i}) ,$ is a tuple of the wards $v_{i} \epsilon V$ visited by the patient at time $t_{i}$. A contact between two patients is established when they are in the same ward at the same time [19]. Mathematically, a contact between two patients is established if any elements of their pathways intersect $P_{m}\cap P_{n} \neq\emptyset$ (*i.e.,* they are in the same ward at the same time). A contact between $N$ patients can be represented by $N\times N$ adjacency matrix $A$ with elements $A_{mn}\neq0$ if two patients were in contact,

$$A_{mn}=\left\{ \begin{aligned} d_{mn}, if P_{m}\cap P_{n} \neq\emptyset\\ 0, otherwirse. \end{aligned} \right.$$

Moreover, non-zero elements of $A$ are weighted by the duration $d_{mn}$ of contact between patients $m$ and $n$. Finally, a weighted contact network $G=(N,E)$ constructed from the adjacency matrix $A$, captures the relation structure of contacts, weighted by duration $E_{mn}= A_{mn}$, between $N$ patients. Movement to other locations for procedures during an inpatient episode, such as endoscopy, interventional radiology or operating theatres, was not examined.

To reveal potential transmission events structure, a patient contact network was constructed from patient movement history, which included information on when and where patients were on the same ward. In this framework, contacts were also weighted by the duration of time spent together across their hospital episode, capturing that the probability of transmission increases with the amount of time two patients spend together [20]. Moreover, a temporal analysis of patient interactions was performed over the time ordered sequence of contacts between patients [21] and used to assess patient roles and position in transmission. All network analysis and visualisation were completed in Python, R, and Cytoscape (cytoscape.org).

# Network community detection

The patient contact network G was clustered to reveal groups of patients more closely linked together using the random walk-based community detection algorithm, Walktrap [22]. In this framework, the weightings of edges (contact duration) directly affect clustering, with the larger edge weights, making two individuals more likely to appear in the same clusters. With the probability of disease transmission increasing with the amount of time two patients spent together [8], this clustering procedure thus highlights patients or groups of patients that are more likely to be involved in the same transmission clusters.

# Correlation analysis

To measure the association between the distance of nodes (patients) in a network and the similarity of those nodes (patient’s microbiology results) in terms of WGS, we correlated the network closeness and the WGS closeness between bacterial isolates. Network closeness was extracted from the contact network by constructing a node-to-node shortest-path matrix, where each entry represented the shortest-path distance from node *i* to node *j*. This shortest-path matrix was then correlated to the matrix of nucleotide-substitution counts extracted from the maximum-likelihood tree of IncHI2 plasmids (Figure 4). Correlation analysis is then employed using a ranked method to account for non-linearities in distances, which we select as Kendall’s rank correlation. Both a high association and significance would be suggestive that the underlying contact network aligns well to the genomic structure indicative of transmission over that network.

# References

1. Otter JA, Mookerjee S, Davies F, *et al*. Detecting carbapenemase-producing Enterobacterales (CPE): an evaluation of an enhanced CPE infection control and screening programme in acute care. *J Antimicrob Chemother*. **2020**; 75(9):2670–2676.

2. Zwaluw K van der, Haan A de, Pluister GN, Bootsma HJ, Neeling AJ de, Schouls LM. The Carbapenem Inactivation Method (CIM), a Simple and Low-Cost Alternative for the Carba NP Test to Assess Phenotypic Carbapenemase Activity in Gram-Negative Rods. *PLOS ONE*. **2015**; 10(3):e0123690.

3. Rodriguez-Manzano J, Moser N, Malpartida-Cardenas K, *et al*. Rapid Detection of Mobilized Colistin Resistance using a Nucleic Acid Based Lab-on-a-Chip Diagnostic System. *Sci Rep*. **2020**; 10(1):8448.

4. Furniss RCD, Dortet L, Bolland W, *et al*. Detection of Colistin Resistance in *Escherichia coli* by Use of the MALDI Biotyper Sirius Mass Spectrometry System. *J Clin Microbiol*. **2019**; 57(12):10.1128/jcm.01427-19.

5. Bolger AM, Lohse M, Usadel B. Trimmomatic: a flexible trimmer for Illumina sequence data. *Bioinformatics*. **2014**; 30(15):2114–2120.

6. Andrews S. FastQC: A quality control tool for high throughput sequence data [Internet]. 2010. Available from: www.bioinformatics.babraham.ac.uk/projects/fastqc

7. Wood DE, Lu J, Langmead B. Improved metagenomic analysis with Kraken 2. *Genome Biol.* **2019**; 20(1):257.

8. Lu J, Breitwieser FP, Thielen P, Salzberg SL. Bracken: Estimating species abundance in metagenomics data. *PeerJ Comput Sci*. **2017**; 2017(1):e104.

9. Clausen PTLC, Aarestrup FM, Lund O. Rapid and precise alignment of raw reads against redundant databases with KMA. *BMC Bioinformatics*. **2018**; 19(1):307.

10. Wick RR, Judd LM, Gorrie CL, Holt KE. Unicycler: Resolving bacterial genome assemblies from short and long sequencing reads. *PLOS Comput Biol*. **2017**; 13(6):e1005595.

11. Seemann T. Abricate [Internet]. GitHub; Available from: https://github.com/tseemann/abricate

12. Jolley KA, Bray JE, Maiden MCJ. Open-access bacterial population genomics: BIGSdb software, the PubMLST.org website and their applications. *Wellcome Open Res*. **2018**; 3:124.

13. Florensa AF, Kaas RS, Clausen PTLC, Aytan-Aktug D, Aarestrup FM. ResFinder – an open online resource for identification of antimicrobial resistance genes in next-generation sequencing data and prediction of phenotypes from genotypes. *Microb Genomics*. **2022**; 8(1):000748.

14. Carattoli A, Zankari E, García-Fernández A, *et al*. *In Silico* Detection and Typing of Plasmids using PlasmidFinder and Plasmid Multilocus Sequence Typing. *Antimicrob Agents Chemother*. **2014**; 58(7):3895–3903.

15. Robertson J, Nash JHE. MOB-suite: software tools for clustering, reconstruction and typing of plasmids from draft assemblies. *Microb Genomics*. **2018**; 4(8).

16. Redondo-Salvo S, Bartomeus-Peñalver R, Vielva L, *et al*. COPLA, a taxonomic classifier of plasmids. *BMC Bioinformatics*. **2021**; 22(1):390.

17. Alikhan N-F, Petty NK, Ben Zakour NL, Beatson SA. BLAST Ring Image Generator (BRIG): simple prokaryote genome comparisons. *BMC Genomics*. **2011**; 12(1):402.

18. Didelot X, Croucher NJ, Bentley SD, Harris SR, Wilson DJ. Bayesian inference of ancestral dates on bacterial phylogenetic trees. *Nucleic Acids Res*. **2018**; 46(22):e134–e134.

19. Meyers L. Contact network epidemiology: Bond percolation applied to infectious disease prediction and control. *Bull Am Math Soc*. **2007**; 44(1):63–86.

20. Gómez S, Arenas A, Borge-Holthoefer J, Meloni S, Moreno Y. Discrete-time Markov chain approach to contact-based disease spreading in complex networks. *Europhys Lett*. **2010**; 89(3):38009.

21. Holme P, Saramäki J. Temporal networks. *Phys Rep*. **2012**; 519(3):97–125.

22. Pons P, Latapy M. Computing Communities in Large Networks Using Random Walks. In: Yolum pInar, Güngör T, Gürgen F, Özturan C, editors. Comput Inf Sci - ISCIS 2005. Berlin, Heidelberg: Springer; 2005. p. 284–293.
